# Social support modifies the association between pre-pregnancy body mass index and breastfeeding initiation in Brazil

**DOI:** 10.1371/journal.pone.0233452

**Published:** 2020-05-21

**Authors:** Mariana Pujól von Seehausen, Rafael Pérez-Escamilla, Maria Inês Couto de Oliveira, Maria do Carmo Leal, Cristiano Siqueira Boccolini

**Affiliations:** 1 Programa de Pós-Graduação em Epidemiologia em Saúde Pública, Escola Nacional de Saúde Pública Sérgio Arouca, Fundação Oswaldo Cruz, Rio de Janeiro, Rio de Janeiro, Brazil; 2 Department of Social and Behavioral Sciences, Yale School of Public Health, New Haven, Connecticut, United States of America; 3 Departamento de Epidemiologia e Bioestatística, Instituto de Saúde Coletiva, Universidade Federal Fluminense, Niterói, Rio de Janeiro, Brazil; 4 Escola Nacional de Saúde Pública Sérgio Arouca, Fundação Oswaldo Cruz, Rio de Janeiro, Rio de Janeiro, Brazil; 5 Laboratório de Informação em Saúde, Instituto de Comunicação e Informação Científica e Tecnológica em Saúde, Fundação Oswaldo Cruz, Rio de Janeiro, Rio de Janeiro, Brazil; University of Mississippi Medical Center, UNITED STATES

## Abstract

**Background:**

Many biological, social and cultural barriers for suboptimal breastfeeding practices have been identified in literature. Among these, excessive pre-pregnancy weight has been identified as a risk factor for not initiating breastfeeding early. Social support, coming from social networks (e.g. a partner, family or friends) or health care providers, has been positively associated with breastfeeding. This study aimed to examine the association between pre-pregnancy excessive weight and breastfeeding within the first hour after birth and if social support modifies this association.

**Design:**

National population-based study conducted with 21,086 postpartum women from February 1, 2011 to October 31, 2012 in 266 hospitals from all five regions of Brazil. Social support was defined as having a companion at the hospital. Main effects and interactions were tested with multivariable regression analyses.

**Results:**

Multivariate regression analyses indicated that class I and class II obese women had lower odds of breastfeeding within the first hour when a companion was not present (AOR = 0.59, 95% CI 0.42–0.82 and AOR = 0.59, 95% CI 0.36–0.97, respectively), but there was no association when the companion was present. Among overweight and obese women, the predicted probability of breastfeeding within the first hour was lower for those without a companion. This association was not found among those with normal pre-pregnancy BMI.

**Conclusions:**

Social support modifies the relationship between pre-gestational BMI and breastfeeding initiation among women who are overweight or obese, specifically it reduces the risk of delayed breastfeeding initiation.

## Introduction

The World Health Organization (WHO) and the United Nations Children’s Fund (UNICEF) recommend that children initiate breastfeeding within the first hour of birth. This recommendation is based on evidence that shows that breastfeeding in the first hour after birth improves the chances of newborn survival and the successful establishment of breastfeeding [1]. In fact, neonates who begin breastfeeding between 2 and 23 hours after birth have a 33 per cent greater risk of dying, and for those who start breastfeeding 24 hours or later after birth, this risk is twofold higher [2,3] compared with those who begin breastfeeding within one hour of birth.

Despite the importance of early initiation of breastfeeding, biological, social, cultural, biomedical and health care systems factors have been identified as barriers for its practice [4–6]. Among these factors, excessive pre-pregnancy weight has been identified as a risk factor for breastfeeding initiation [7–11]. These findings are highly relevant for Brazil, where 48.7% of women of reproductive age are overweight or obese [12].

Social support, which is a key factor underlying social relationships, can be broadly classified into four categories: emotional (empathy, love, trust and care), instrumental (tangible aid and services that directly assist a person in need), informational (advice, suggestions and information that a person can use to address problems) and appraisal (constructive feedback) [13]. In Brazil, the right to have continuous support during childbirth is supported by law and it is usually offered by women’s partners, mothers, siblings or friends [14]. Social support, coming from social networks (e.g. a partner, family or friends) [15] or health care providers [16], has been positively associated with breastfeeding. Hence, we hypothesized that social support may protect mothers with excessive pre-pregnancy weight from having a delayed breastfeeding initiation.

The aim of this study was to explore the association between pre-pregnancy excessive weight and breastfeeding within the first hour after birth, and to determine if social support modifies this association, using data from the nationally representative Birth in Brazil Study, which among other things was designed to identify risk factors for breastfeeding failure among obese women. Therefore we expect that this study will contribute to fill in a major gap in the literature [17].

## Methods

The data for the present study were obtained from the Birth in Brazil study, which is a national population-based study of postpartum women and their newborns carried out from February 1, 2011 to October 31, 2012. It included 23,894 women interviewed in 266 hospitals from all five regions of Brazil [18].

### Sampling and data collection

The Birth in Brazil study had a probabilistic and complex three-stage sampling design. In the first stage, all hospitals which had 500 or more births per year in 2007 were identified, stratified by Region (North, Northeast, Southeast, Central-West and South), municipality (state capital or interior) and type of hospital (private, public or mixed) which were selected with probability proportional to size (PPS), defined by number of live births at each hospital. In each stratum, at least five hospitals and 450 women (a total of 90 women per hospital) were selected. Sample size was estimated based on the caesarean section rate in Brazil in 2007 of 46.6% to detect a difference between hospitals of at least 14%, an alpha of 5% and power of 95%. In the second stage, an inverse sampling method was used to determine the number of days needed to interview 90 puerperal women in each hospital. To account for the difference of number of live births in weekends and work days, this period had to be of at least seven days in each hospital to ensure the recruitment of representative samples. In the final stage, the women eligible on each day of the fieldwork were selected. A total of 1,356 (5.7%) of women in the original sample were replaced, 15% due to early hospital discharge and 85% due to refusal to participate [18].

All women who had given birth to a live newborn, regardless of weight or gestational age, or to a stillbirth with birth weight ≥ 500 g and/or gestational age ≥ 22 weeks of pregnancy in one of the sampled hospitals during the data collection were invited to participate. All women with miscarriages were excluded. In total, 23,894 women were interviewed at 266 hospitals distributed across 191 municipalities, covering all the 27 Brazilian states. Post-partum women were interviewed face-to-face, within the first 24 hours after birth, to collect information on breastfeeding practices, socio-economic, demographic, biomedical as well as prenatal, delivery care, and pregnancy outcome indicators. Hospital directors were also interviewed to collect information on the hospital characteristics such as hospital funding sources and Baby-Friendly Hospital Initiative accreditation status. Further details on the data collection and the sample design are reported elsewhere [18,19].

### Data analysis

Exclusion criteria for our analytical sample included the following conditions that may impede breastfeeding: mothers who tested positive for HIV [20], mothers or babies who were too sick to initiate breastfeeding [21], neonatal death, gestational age < 34 weeks, infant malformation [22], resulting in a final sample of 21,086 maternal-infant dyads.

### Variables

The main exposure (pre-pregnancy body mass index (BMI)), the outcome (timing of breastfeeding initiation) and the following potential confounders were selected based on findings from previous studies [4–6]. Socio-economic and demographic variables included maternal age (12–19 years, 20–34 years, ≥35 years); educational level (up to Elementary school (<11 years); High school or more (≥ 11 years); parity (primiparous; multiparous); and geographical region (North; Northeast; Southeast; Central-West; South). Health care system factors included maternal receipt of breastfeeding during prenatal care (yes; no); combination of hospital funding source and Baby-Friendly Hospital (BFH) status (public and BFH; mixed and BFH; private and BFH; public and not BFH; mixed and not BFH; private and not BFH); and type of delivery (vaginal; antepartum cesarean, defined as the cesarean section with no spontaneous or induction of labor; intrapartum cesarean, defined as the cesarean section performed during labor). The independent or exposure variable pre-pregnancy BMI was coded into six mutually exclusive categories (underweight; normal weight; overweight; class I obese; class II obese; class III obese). Pre-pregnancy BMI, was calculated using height and weight measurements collected either from prenatal care cards (30.1% and 35.4%, respectively) or from the interviews with the mothers (54.7% and 60.5%, respectively). Heights were missing for 15.2% and weights for 4.0% of the mothers. The combination of missing information in both variables led to a total of 17.2% missing cases in the calculated pre-pregnancy BMI. For these cases, we imputed BMI following the Multivariate Imputation by Chained Equations (MICE) [23] approach with the ‘mi’ module from the statistical software STATA (version 15.0). The BMI cut-off points used were based on those recommended by the World Health Organization (WHO) [24]: underweight (<18.5 kg/m^2^), normal weight (18.5–24.9 kg/m^2^), overweight (25–29.9 kg/m^2^), class I obesity (30–34.9 kg/m^2^), class II obesity (35–39.9 kg/m^2^) and class III obesity (>40 kg/m^2^).

The outcome, breastfeeding within the first hour after birth, was based on maternal self-report and categorized as a dichotomous variable (yes, no). Research staff queried the mother within the first 24 hours after birth regarding breastfeeding in the delivery room and the time at which breastfeeding was initiated.

Social support was defined as having a companion at the hospital during any or all moments and categorized in a dichotomous variable (yes, no), based on questions asked to the mother by research staff.

Information regarding hospital funding was provided by the hospital directors: public hospitals are financed exclusively with public funds; private hospitals receive only private funds (direct payment or health insurance reimbursement) and mixed hospitals are private institutions which receive public funds for a percentage of the hospital admissions.

### Multivariable statistical analyses

The statistical analyses were performed with Stata/SE software version 15. Given the complex sampling design, we used the ‘svy’ module from Stata (version 15.0) to conduct univariate and multiple main effects and interaction regression analyses between the outcome (early breastfeeding initiation) and the main exposure (maternal BMI category), adjusting for covariates. Covariates with a significance level less than 0.20 were selected for multiple logistic regression models. The interaction between pre-pregnancy body mass index and social support (i.e. have a companion or not) at the hospital was also included in the model, to test the original hypothesis that social support may protect mothers with excessive pre-pregnancy weight from a delay in breastfeeding initiation. This interaction was found to be statistically significant (p<0.001). Hence, we used the model with the interaction term to calculate the predicted probabilities of breastfeeding within the first hour for each BMI category [25]. Furthermore, the final models were ran stratifying the sample by social support status category. Regression findings were expressed as Adjusted Odds Ratios (AOR) and their corresponding 95% confidence intervals (95% CIs). Because only 0.5% of women (N = 91; N = 71 with social support, N = 20 without social support) fell in the obese class III category, regression estimates were very unstable, i.e., with very wide confidence intervals, especially for the models representing the subsample of women without social support. Therefore, this subgroup of women was excluded from the hypothesis testing multivariable statistical analyses.

### Details of ethics approval

This study was carried out in accordance with the Brazilian National Health Council Resolution n. 196/96. The ethics committee of the Sérgio Arouca National School of Public Health, Oswaldo Cruz Foundation (CEP/ENSP), approved the Birth in Brazil study on May 11th 2010 under the research protocols CAAE: 0096.0.031.000–10. All hospital directors and postpartum women provided signed informed consent.

## Results

The prevalence of early breastfeeding initiation was 58.1%. More than half of the mothers had a BMI within the normal range, 7% were underweight, 21.0% were overweight, 6% had class I, 1.9% had class II and 0.5% had class III obesity. Approximately one fifth of the mothers were adolescents and more than half had elementary school education or less. Almost 65% obtained information about the importance of breastfeeding during prenatal visits. Approximately one fourth of the mothers had their babies at public Baby-Friendly hospitals. The prevalence of antepartum cesarean section was 42.8%. The descriptive characteristics varied between those who had and those who did not have in-hospital social support. Among those with social support, 55.6% had more than high school education, compared to 36.1% of those without social support. While one quarter of the mothers with social support had their babies in private non-Baby-Friendly hospitals; less than 3% of the mothers without social support had their babies in these hospitals. Furthermore, more than half of the mothers who had social support had their babies through antepartum cesarean, while more than three quarters of those who did not have social support had their babies through vaginal delivery (Table 1).

**Table 1:** Prevalence of breastfeeding within the first hour after birth and maternal, hospital and child characteristics by social support status, Brazil, 2011.

	All Respondents		With social support		Without social support	
Variables	n	%	n	%	n	%
**Breastfeeding within the first hour**						
Yes	11,777	58.3	9025	58.2	2747	58.4
No	8,432	41.7	6478	41.8	1952	41.6
**Maternal age**						
12–19 years	3,839	19.2	2,890	19.2	949	19.1
20–34 years	14,983	70.8	11,369	70.5	3,614	72.0
35 years or more	2,264	9.9	1,816	10.3	448	8.9
**Educational level**						
Up to Elementary school (<11 years of schooling)	10,343	52.2	7,138	44.4	3,205	63.9
High school or more (≥11 years of schooling)	10,743	47.8	8,937	55.6	1,806	36.1
**Parity**						
Multiparous	9,826	46.6	8,127	50.6	1,699	33.9
Primiparous	11,260	53.4	7,948	49.4	3,312	66.1
**Region of residence**						
North	2,593	9.8	1,973	9.4	620	10.8
Northeast	5,429	29.0	3,851	28.4	1,578	30.5
Southeast	6,992	42.3	5,543	43.5	1,449	38.5
South	3,575	12.4	2,944	13.5	631	9.8
Middle West	2,497	6.6	1,764	5.4	733	10.3
**Information about breastfeeding at prenatal care**						
Yes	13,901	66.9	10,755	67.6	3,146	64.5
No	6,889	33.1	5,155	32.4	1,734	35.5
**Pre-pregnancy BMI**						
Underweight (<18.5 kg/m^2^)	1,331	7.6	976	7.3	355	8.9
Normal weight (18.5–24.9 kg/m^2^)	10,868	62.3	8,448	62.6	2420	61.0
Overweight (25.0–29.9 kg/m^2^)	3,667	21.0	2,859	21.2	808	20.4
Class I obesity (30–34.9 kg/m^2^)	1,177	6.7	895	6.6	282	7.1
Class II obesity (35–39.9 kg/m^2^)	326	1.9	244	1.8	82	2.1
Class III obesity (>40 kg/m^2^)	91	0.5	71	0.5	20	0.5
**Hospital**						
Public Baby-Friendly	4,007	23.5	2,695	16.8	1,312	26.2
Public non-Baby-Friendly	3,283	16.3	2,077	12.9	1,206	24.1
Mixed Baby-Friendly	3,350	15.5	2,695	16.8	655	13.1
Mixed non-Baby-Friendly	5,907	29.9	4,229	26.3	1,678	33.5
Private Baby-Friendly	237	0.8	222	1.3	15	0.3
Private non-Baby-Friendly	4,302	14.0	4,157	25.9	145	2.8
**Type of delivery**						
Vaginal	10,061	48.9	6,198	38.6	3,863	77.1
Intrapartum caesarean	1,727	8.3	1,313	8.2	414	8.3
Antepartum caesarean	9,298	42.8	8,564	53.3	734	14.6

Early breastfeeding initiation was more common among adolescents, mothers who completed elementary school, multiparous women and those who lived in the North region. Rates of early breastfeeding initiation were also higher among women who obtained prenatal information about the importance of breastfeeding. Women who gave birth at Baby-Friendly hospitals, regardless of their funding sources, and those who delivered vaginally also had higher early breastfeeding initiation rates. Bivariate analysis also indicated that, compared to mothers with normal BMI, overweight and class I obese women were less likely to initiate breastfeeding early. Furthermore, stratified analyses showed that obese women were less likely to initiate breastfeeding early only when social support was not available (Table 2).

**Table 2:** Unadjusted factors associated with breastfeeding in the first hour after birth, according to mother, newborn and hospital characteristics by social support status, Brazil, 2011.

	All Respondents		With social support		Without social support	
Variables	Unadjusted OR	CI 95%	Unadjusted OR	CI 95%	Unadjusted OR	CI 95%
**Maternal age**						
12–19 years	1.92	1.61–2.30	2.04	1.67–2.49	1.57	1.16–2.14
20–34 years	1.50	1.31–1.71	1.55	1.34–1.80	1.31	1.04–1.66
35 years or more	1.0		1.0		1.0	
**Educational level**						
Up to Elementary school (<11 years of schooling)	1.66	1.48–1.86	1.79	1.57–2.03	1.35	1.14–1.61
High school or more (≥11 years of schooling)	1.0		1.0		1.0	
**Parity**						
Multiparous	1.27	1.16–1.39	1.31	1.19–1.45	1.16	0.99–1.35
Primiparous	1.0		1.0		1.0	
**Region of residence**						
North	2.39	1.73–3.31	2.31	1.68–3.17	2.73	1.46–5.12
Northeast	1.08	0.81–1.45	0.97	0.70–1.32	1.57	1.01–2.42
Southeast	1.0		1.0		1.0	
South	1.45	0.94–2.23	1.45	0.94–2.23	1.44	0.75–2.77
Middle West	1.58	1.12–2.20	1.48	1.02–2.14	1.90	1.13–3.18
**Information about breastfeeding at prenatal care**						
Yes	1.27	1.13–1.43	1.26	1.11–1.42	1.32	1.07–1.62
No	1.0		1.0		1.0	
**Pre-pregnancy BMI**						
Underweight (<18.5 kg/m^2^)	1.25	1.05–1.49	1.31	1.09–1.58	0.92	0.66–1.30
Normal weight (18.5–24.9 kg/m^2^)	1.0		1.0		1.0	
Overweight (25.0–29.9 kg/m^2^)	0.86	0.77–0.95	0.91	0.81–1.02	0.77	0.61–0.99
Class I obesity (30–34.9 kg/m^2^)	0.75	0.63–0.89	0.84	0.68–1.05	0.56	0.39–0.80
Class II obesity (35–39.9 kg/m^2^)	0.80	0.60–1.07	0.98	0.68–1.41	0.53	0.33–0.84
Class III obesity (>40 kg/m^2^)	1.05	0.59–1.87	0.98	0.46–2.08	1.25	0.36–4.22
**Hospital**						
Public Baby-Friendly	5.34	3.65–7.82	5.64	3.83–8.29	6.79	2.71–17.0
Public non-Baby-Friendly	3.52	2.34–5.28	3.65	2.35–5.65	4.81	1.93–11.9
Mixed Baby-Friendly	4.57	3.09–6.76	4.91	3.32–7.26	5.05	1.13–7.39
Mixed non-Baby-Friendly	2.16	1.42–3.29	2.24	1.45–3.44	2.90	1.14–7.38
Private Baby-Friendly	6.72	3.57–12.6	7.02	3.85–12.7	4.56	0.78–26.3
Private non-Baby-Friendly	1.0		1.0		1.0	
**Type of delivery**						
Vaginal	3.16	2.68–3.72	3.27	2.72–3.91	3.27	2.55–4.18
Intrapartum caesarean	1.29	1.09–1.53	1.38	1.15–1.67	1.09	0.80–1.48
Antepartum caesarean	1.0		1.0		1.0	

**Fig 1:**
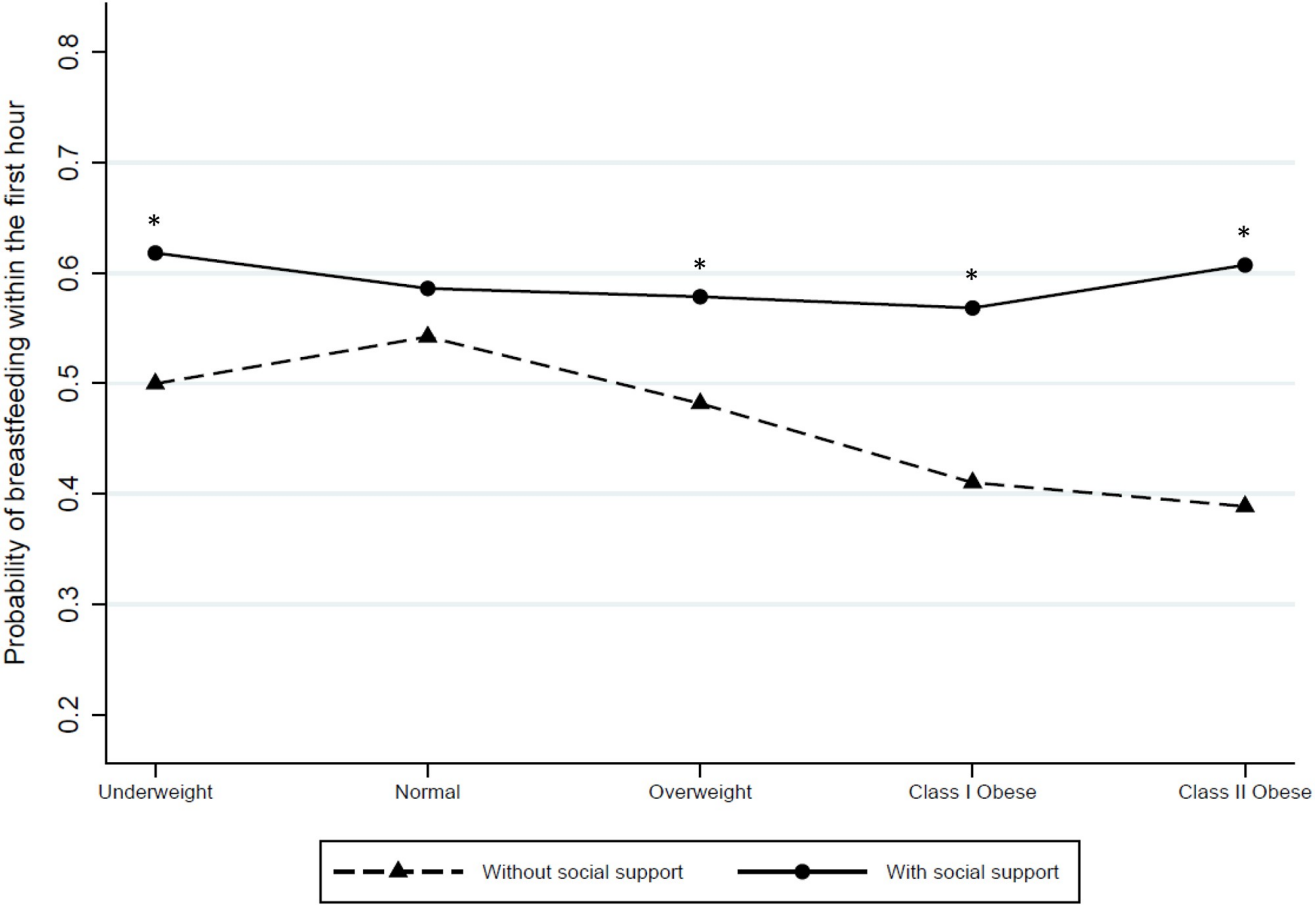
Predicted probabilities of breastfeeding within the first hour according to pre-gestational BMI and social support status. * Denotes subgroups statistical differences.

Multivariate regression analyses indicated that class I and class II obese women had lower odds of breastfeeding within the first hour when social support was not provided (Table 3). Accordingly, the predicted probability of breastfeeding within the first hour was lower for those without social support. By contrast this association was not found for mothers with normal pre-pregnancy BMI. Thus, these results suggest that social support can protect mothers who have excessive body weight against a delay in breastfeeding initiation (Fig 1).

**Table 3:** Final adjusted models for the association between pre-pregnancy BMI and breastfeeding in the first hour after birth by social support status, Brazil, 2011.

Variables	With social support		Without social support	
	Model 1		Model 2	
	Adjusted OR^a^	CI 95%	Adjusted OR^a^	CI 95%
**Pre-pregnancy BMI**				
Underweight	1.12	0.92–1.36	0.86	0.61–1.20
Normal	1.0		1.0	
Overweight	0.94	0.83–1.07	0.83	0.65–1.07
Class I Obese	0.91	0.72–1.15	0.59	0.42–0.82
Class II Obese	1.08	0.76–1.53	0.59	0.36–0.97

^a^ Adjusted OR: odds ratio adjusted by logistic regression for maternal age, parity, region, receiving information about breastfeeding during prenatal care, hospital funding and Baby-Friendly Hospital Initiative status, type of delivery.

## Discussion

More than half of the newborns from this nationally representative study were breastfed within the first hour after birth, as previously reported [4]. However, the prevalence of this outcome varied according to pre-pregnancy BMI: class I and class II obese mothers were less likely to initiate breastfeeding within the recommended time. Similar findings have been reported in other countries [9–11] and in a small-scale study in Brazil [26], yet this is the first study to investigate the association between pre-pregnancy BMI and breastfeeding initiation and whether or not this relationship is modified by social support.

Our adjusted findings suggest that social support may protect obese mothers against a delay in breastfeeding initiation. The presence of a companion has previously been reported as positively associated with breastfeeding practices [16]. In fact, research conducted in Nigeria found that social support during childbirth acted as a catalyst for early breastfeeding initiation: the median time to initiate breastfeeding was shorter for mothers who had companions [15,27]. We hypothesize that it is possible that companions provide psychological and possibly physical support during labor, delivery and the postpartum period.

Existing literature has suggested multiple mechanisms through which obesity can lead to a delay in breastfeeding initiation, including psychological ones. It is possible that obese women may have lower self-esteem, greater body image dissatisfaction, and poorer mental health compared to non-obese women [7]. Indeed, women with increased concerns about their body shape or weight are less likely to intend to breastfeed [7]. Hauff *et al*. [28] showed that, although obese women intended to breastfeed as much as non-obese women, there were psychosocial risk factors that may have explained, at least in part, the lower prevalence of breastfeeding initiation and shorter breastfeeding duration among them. Indeed, once statistical models were adjusted for these psychological factors, BMI was no longer significantly associated with either of these breastfeeding outcomes. The psychosocial risk factors included lower confidence that they would reach their breastfeeding goals, reporting fewer close friends or relatives who had breastfed, and lower social influence from others to breastfeed. All of these factors have the potential to trigger or exacerbate stress, which in turn has been associated with impaired lactogenesis [29]. In our study, social support (proxied by the presence of a companion in the maternity hospital) may have helped reduce stress [30] among obese women and partly explained how it protected against the risk of not initiating breastfeeding early.

The physiological mechanism underlying the relationship between social support, stress and breastfeeding may be linked to oxytocin, a neuropeptide that has stress-attenuating and anxiolytic effects and is also necessary for the milk ejection reflex. Suckling stimulates the posterior pituitary gland to release oxytocin, which causes the contraction of the myoepithelial cells surrounding the alveoli, forcing milk from the alveoli into the milk ducts [31]. In obese women, the effect of oxytocin may be lessened by higher levels of leptin, which is a hormone secreted by adipose tissue and has been shown to inhibit oxytocin activity on human muscle cells in vitro [32]. The beneficial effects of social interaction on stress reduction seem to be associated with oxytocin as it has been shown that receiving positive social interactions triggers a release of this neuropeptide. For instance, positive communication and physical contact between married couples are associated with higher plasma oxytocin levels [33], which can dampen physiological stress levels. It is possible that higher plasma oxytocin levels are associated with low norepinephrine levels, blood pressure and heart rate [34]. Therefore, social support may protect overweight and obese mothers against a delay in breastfeeding initiation by triggering oxytocin release and possibly compensating for the potential inhibiting effect of leptin on human lactation.

A third possible explanation for the lower prevalence of early breastfeeding initiation among obese women is the possibility that it may be difficult for the baby to attach to the breast, as a result of additional body tissue, larger areolas and larger breasts that reduce lap area [17]. In this scenario, social support may have positively affected breastfeeding by being a source of hands-on support. As a Cochrane systematic review showed, although all forms of extra support had a positive impact on breastfeeding outcomes, strategies that rely on face-to-face support are more likely to succeed [16], possibly because they may include some form of physical support. This form of extra support may be even more relevant for obese women, as a study in the USA showed that obese mothers had lower odds of being exposed to hospital practices associated with improved breastfeeding outcomes, including being given breastfeeding help by a staff member. In that study, many providers expressed that they disliked or even dreaded providing postpartum care for obese women, as it required extra work [35].

Findings from our study have important longer term implications as evidence suggests that early breastfeeding initiation may protect obese mothers from failure to breastfeed for longer. Kair *et al*. [36] showed that obese mothers had three times greater odds of exclusively breastfeeding at one week if they reported exposure to the fourth step of the Ten Steps of Successful Breastfeeding; i.e. uninterrupted skin-to-skin contact since birth and support for mothers to initiate breastfeeding within one hour after birth. Moreover, exclusive in-hospital formula-feeding is a risk factor for delayed onset of lactation [37].

Other covariates associated with breastfeeding initiation within the first hour were maternal age, parity, geographical region, receiving information about breastfeeding during prenatal care, hospital funding sources combined with BFHI accreditation status, and type of delivery. These findings are consistent with previous studies [4].

Our findings documented a negative dose response relationship between pre-pregnancy BMI and early breastfeeding initiation among those without social support. However, among those with social support, obesity was not a risk factor for delayed breastfeeding initiation. From a public health perspective, our findings call for supporting initiatives allowing the presence of a companion during childbirth, paying special attention to the psycho-emotional and biomedical needs of women with excessive body weight.

Previous interventions focusing on increasing breastfeeding duration and exclusivity in obese women have not been successful [17]. Interestingly, none of them focused on strengthening the supportive role from companions. Moreover, breastfeeding within the first hour of life was not explored in any of the studies. Therefore, it is also important to design and test interventions that provide additional support to breastfeeding women with excessive body weight for success with early breastfeeding initiation and their longer term breastfeeding plans. Such interventions should consider including professional as well as birth companion’s social and breastfeeding support.

One limitation of our study was the use of anthropometric data that was not directly measured by the research team. Likewise, the outcome was based on maternal self-report and was not directly observed by the research group, possibly leading to recall bias. However, despite these limitations, our findings are based on a national survey with countrywide representation. To our knowledge, this is the first study to show a buffering effect of social support on the relationship between pre-pregnancy BMI and breastfeeding within the first hour of life among women with excessive body weight.

## Conclusion

Consistent with other studies, our findings show that obese mothers are less likely to breastfeed after birth. Although the mechanisms by which breastfeeding initiation is affected by excessive weight are not clear, our study indicates that social support may play an important role in this relationship by promoting breastfeeding initiation. Our findings call for further exploring how best to design interventions that include in-hospital companions to help obese mothers to successfully initiate breastfeeding within one hour after birth.

## Supporting information

S1 Table Full results of the final adjusted models for the association between pre-pregnancy BMI and breastfeeding in the first hour after birth by social support status, Brazil, 2011. (DOCX)

S2 Table Full results of the predicted probabilities of breastfeeding within the first hour by pre-gestational BMI and social support status, Brazil, 2011. (DOCX)

## References

[1] World Health Organization. Guideline: protecting, promoting and supporting breastfeeding in facilities providing maternity and newborn services. Geneva: World Health Organization; 2009. https://apps.who.int/iris/bitstream/handle/10665/259386/9789241550086-eng.pdf?sequence=1 29565522

[2] Smith ER, Hurt L, Chowdhury R, Sinha B, Fawzi W, Edmond KM. Delayed breastfeeding initiation and infant survival: A systematic review and meta-analysis. PLoS One. 2017 7 26;12(7):e0180722 10.1371/journal.pone.0180722 28746353 PMC5528898

[3] Boccolini CS, Carvalho ML, Oliveira MI, Pérez-Escamilla R. Breastfeeding during the first hour of life and neonatal mortality. J Pediatr (Rio J). 2013 Mar-Apr;89(2):131–6. 23642422 10.1016/j.jped.2013.03.005

[4] Carvalho ML, Boccolini CS, Oliveira MI, Leal MD. The baby-friendly hospital initiative and breastfeeding at birth in Brazil: a cross sectional study. Reprod Health. 2016 10 17;13(Suppl 3):119 10.1186/s12978-016-0234-9 27766969 PMC5073809

[5] Esteves TM, Daumas RP, Oliveira MI, Andrade CA, Leite IC. Factors associated to breastfeeding in the first hour of life: systematic review. Rev Saude Publica. 2014 8;48(4):697–708. 10.1590/S0034-8910.2014048005278 25210829 PMC4181097

[6] Boccolini CS, Carvalho ML, Oliveira MI, Vasconcellos AG. Factors associated with breastfeeding in the first hour of life. Rev Saude Publica. 2011 2;45(1):69–78. Epub 2010 Nov 12. 10.1590/s0034-89102010005000051 21085886

[7] Amir LH, Donath S. A systematic review of maternal obesity and breastfeeding intention, initiation and duration. BMC Pregnancy Childbirth. 2007 7 4;7:9 10.1186/1471-2393-7-9 17608952 PMC1937008

[8] Li R, Jewell S, Grummer-Strawn L. Maternal obesity and breastfeeding practices. Am J Clin Nutr. 2003 4;77(4):931–6. 10.1093/ajcn/77.4.931 12663294

[9] Sebire NJ, Jolly M, Harris JP, Wadsworth J, Joffe M, Beard RW et al Maternal obesity and pregnancy outcome: a study of 287,213 pregnancies in London. Int J Obes Relat Metab Disord. 2001 8;25(8):1175–82. 10.1038/sj.ijo.0801670 11477502

[10] Donath SM, Amir LH. Does maternal obesity adversely affect breastfeeding initiation and duration? J Paediatr Child Health. 2000 10;36(5):482–6. 10.1046/j.1440-1754.2000.00562.x 11036806

[11] Martinez JL, Chapman DJ, Pérez-Escamilla R. Prepregnancy Obesity Class Is a Risk Factor for Failure to Exclusively Breastfeed at Hospital Discharge among Latinas. J Hum Lact. 2016 5;32(2):258–68. 10.1177/0890334415622638 26747829

[12] Ministério da Saúde do Brasil. Pesquisa Nacional de Saúde 2013. Rio de Janeiro: IBGE, 2015 ftp://ftp.ibge.gov.br/PNS/2013/pns2013.pdf

[13] Heany CA, Israel BA. Social networks and social support In: Glanz K, Rimer BK and Viswanath K, editors. Health Behavior and Health Education. San Francisco: Jossey-Bass; 2008, 4th ed, pp 189–207.

[14] Diniz CS, d’Orsi E, Domingues RM, Torres JA, Dias MA, Schneck CA et al Implementation of the presence of companions during hospital admission for childbirth: data from the Birth in Brazil national survey. Cad Saude Publica. 2014 8;30 Suppl 1:S1–14. 10.1590/0102-311x00127013 25167174

[15] Morhason-Bello IO, Adedokun BO, Ojengbede OA, Olayemi O, Oladokun A, Fabamwo AO. Assessment of the effect of psychosocial support during childbirth in Ibadan, south-west Nigeria: a randomised controlled trial. Aust N Z J Obstet Gynaecol. 2009 4;49(2):145–50. 10.1111/j.1479-828X.2009.00983.x 19432601

[16] McFadden A, Gavine A, Renfrew MJ, Wade A, Buchanan P, Taylor JL. Support for healthy breastfeeding mothers with healthy term babies. Cochrane Database Syst Rev. 2017 2 28;2:CD001141 10.1002/14651858.CD001141.pub5 28244064 PMC6464485

[17] Bever Babendure J, Reifsnider E, Mendias E, Moramarco MW, Davila YR. Reduced breastfeeding rates among obese mothers: a review of contributing factors, clinical considerations and future directions. Int Breastfeed J. 2015 7 1;10:21 10.1186/s13006-015-0046-5 26140049 PMC4488037

[18] Vasconcellos MT, Silva PL, Pereira AP, Schilithz AO, Souza Junior PR, Szwarcwald CL. Sampling design for the Birth in Brazil: National Survey into Labor and Birth. Cad Saude Publica. 2014 8;30 Suppl 1:S1–10. 10.1590/0102-311x00176013 25167189

[19] do Carmo Leal M, da Silva AA, Dias MA, da Gama SG, Rattner D, Moreira ME et al Birth in Brazil: national survey into labour and birth. Reprod Health. 2012 8 22;9:15 10.1186/1742-4755-9-15 22913663 PMC3500713

[20] Domingues RM, Szwarcwald CL, Souza PR Jr, Leal Mdo C. Prenatal testing and prevalence of HIV infection during pregnancy: data from the "Birth in Brazil" study, a national hospital-based study. BMC Infect Dis. 2015 2 26;15:100 10.1186/s12879-015-0837-8 25880460 PMC4346116

[21] Dias MA, Domingues RM, Schilithz AO, Nakamura-Pereira M, Diniz CS, Brum IR. Incidence of maternal near miss in hospital childbirth and postpartum: data from the Birth in Brazil study. Cad Saude Publica. 2014 8;30 Suppl 1:S1–12. 10.1590/0102-311x00154213 25167176

[22] Silva AA, Leite AJ, Lamy ZC, Moreira MEL, Gurgel RQ, Cunha AJLA. Morbidade neonatal near miss na pesquisa Nascer no Brasil. Cad Saude Publica. 2014 30(Suppl 1):S182–S191. 10.1590/0102-311x00129613 25167178

[23] Azur MJ, Stuart EA, Frangakis C, Leaf PJ. Multiple imputation by chained equations: what is it and how does it work? Int J Methods Psychiatr Res. 2011 3;20(1):40–9. 10.1002/mpr.329 21499542 PMC3074241

[24] World Health Organization. Physical status: the use and interpretation of anthropometry. Report of a WHO Expert Committee. WHO Technical Report Series 854. Geneva: World Health Organization, 1995. https://apps.who.int/iris/bitstream/handle/10665/37003/WHO_TRS_854.pdf?sequence=1 8594834

[25] Kollannoor-Samuel G, Wagner J, Damio G, Segura-Pérez S, Chhabra J, Vega-López S et al Social support modifies the association between household food insecurity and depression among Latinos with uncontrolled type 2 diabetes. J Immigr Minor Health. 2011 12;13(6):982–9. 10.1007/s10903-011-9499-9 21789561 PMC3205303

[26] Pinheiro TV, Goldani MZ; IVAPSA group. Maternal pre-pregnancy overweight/obesity and gestational diabetes interaction on delayed breastfeeding initiation. PLoS One. 2018 6 18;13(6):e0194879 10.1371/journal.pone.0194879 29912885 PMC6005508

[27] Morhason-Bello IO, Adedokun BO, Ojengbede OA. Social support during childbirth as a catalyst for early breastfeeding initiation for first-time Nigerian mothers. Int Breastfeed J. 2009 12 10;4:16 10.1186/1746-4358-4-16 20003310 PMC2799385

[28] Hauff LE, Leonard SA, Rasmussen KM. Associations of maternal obesity and psychosocial factors with breastfeeding intention, initiation, and duration. Am J Clin Nutr. 2014 3;99(3):524–34. 10.3945/ajcn.113.071191 24401717 PMC3927688

[29] Dewey KG. Maternal and fetal stress are associated with impaired lactogenesis in humans. J Nutr. 2001 11;131(11):3012S–5S. 10.1093/jn/131.11.3012S 11694638

[30] Cohen S. Social Relationships and Health. Am Psychol. 2004 11;59(8):676–684. 10.1037/0003-066X.59.8.676 15554821

[31] Ramsay DT, Kent JC, Owens RA, Hartmann PE. Ultrasound imaging of milk ejection in the breast of lactating women. Pediatrics. 2004 2;113(2):361–7. 10.1542/peds.113.2.361 14754950

[32] Moynihan AT, Hehir MP, Glavey SV, Smith TJ, Morrison JJ. Inhibitory effect of leptin on human uterine contractility in vitro. Am J Obstet Gynecol. 2006 8;195(2):504–9. 10.1016/j.ajog.2006.01.106 16647683

[33] Gordon I, Martin C, Feldman R, Leckman JF. Oxytocin and social motivation. Developmental cognitive neuroscience. 2011;1:471–493. 10.1016/j.dcn.2011.07.007 21984889 PMC3185363

[34] Olff M, Frijling JL, Kubzansky LD, Bradley B, Ellenbogen MA, Cardoso C et al The role of oxytocin in social bonding, stress regulation and mental health: an update on the moderating effects of context and interindividual differences. Psychoneuroendocrinology. 2013 9;38(9):1883–94. 10.1016/j.psyneuen.2013.06.019 23856187

[35] Kair LR, Colaizy TT. Obese Mothers have Lower Odds of Experiencing Pro-breastfeeding Hospital Practices than Mothers of Normal Weight: CDC Pregnancy Risk Assessment Monitoring System (PRAMS), 2004–2008. Matern Child Health J. 2016 3;20(3):593–601. 10.1007/s10995-015-1858-z 26515471

[36] Kair LR, Nickel NC, Jones K, Kornfeind K, Sipsma HL. Hospital Breastfeeding Support and Exclusive Breastfeeding by Maternal Pre-Pregnancy BMI. Matern Child Nutr. 2019 7;15(3):e12783 10.1111/mcn.12783 30659747 PMC6594899

[37] Chapman DJ, Pérez-Escamilla R. Identification of risk factors for delayed onset of lactation. J Am Diet Assoc. 1999 4;99(4):450–4; quiz 455–6. 10.1016/S0002-8223(99)00109-1 10207398

