# Supplementary material for: Social support modifies the association between pre-pregnancy body mass index and breastfeeding initiation in Brazil
Source: PLoS One. 2020 May 21;15(5):e0233452. doi: 10.1371/journal.pone.0233452 (PMC7242016; doi:10.1371/journal.pone.0233452)
Supplement: S1 Table — (DOCX) [file pone.0233452.s001.docx]

S1 Table. Full results of the final adjusted models for the association between pre-pregnancy BMI and breastfeeding in the first hour after birth according to social support status, Brazil, 2011.

|  | **Adjusted OR^a^** | **Standard error** | **p-value** | **95% CI** |
| --- | --- | --- | --- | --- |
| **With social support** |  |  |  |  |
| Underweight | 1.12 | 0.109 | 0.242 | 0.92-1.36 |
| Normal | 1.0 |  |  |  |
| Overweight | 0.94 | 0.059 | 0.355 | 0.83-1.07 |
| Class I Obese | 0.91 | 0.106 | 0.423 | 0.72-1.15 |
| Class II Obese | 1.08 | 0.189 | 0.657 | 0.76-1.53 |
| **Without social support** |  |  |  |  |
| Underweight | 0.86 | 0.146 | 0.374 | 0.61-1.20 |
| Normal | 1.0 |  |  |  |
| Overweight | 0.83 | 0.106 | 0.156 | 0.65-1.07 |
| Class I Obese | 0.59 | 0.101 | 0.002 | 0.42-0.82 |
| Class II Obese | 0.59 | 0.147 | 0.036 | 0.36-0.97 |

^a^ Adjusted by logistic regression for maternal age, parity, region, receiving information about breastfeeding during prenatal care, hospital funding and accreditation to the Baby-Friendly Hospital Initiative and type of delivery.
