# Supplementary material for: Social support modifies the association between pre-pregnancy body mass index and breastfeeding initiation in Brazil
Source: PLoS One. 2020 May 21;15(5):e0233452. doi: 10.1371/journal.pone.0233452 (PMC7242016; doi:10.1371/journal.pone.0233452)
Supplement: S2 Table — (DOCX) [file pone.0233452.s002.docx]

S2 Table. Full results of the predicted probabilities of breastfeeding within the first hour according to pre-gestational BMI and social support status.

|  | **Predicted probability** | **Standard error** | **95% CI** | **p-value^a^** |
| --- | --- | --- | --- | --- |
| **Underweight** |  |  |  |  |
| With social support | 0.61 | 0.038 | 0.57-0.66 | 0.003 |
| Without social support | 0.49 | 0.024 | 0.42-0.56 |  |
| **Normal weight** |  |  |  |  |
| With social support | 0.58 | 0.017 | 0.55-0.62 | 0.08 |
| Without social support | 0.54 | 0.026 | 0.49-0.59 |  |
| **Overweight** |  |  |  |  |
| With social support | 0.58 | 0.019 | 0.54-0.62 | 0.003 |
| Without social support | 0.48 | 0.030 | 0.42-0.53 |  |
| **Class I obesity** |  |  |  |  |
| With social support | 0.57 | 0.019 | 0.52-0.61 | <0.001 |
| Without social support | 0.41 | 0.039 | 0.33-0.48 |  |
| **Class II obesity** |  |  |  |  |
| With social support | 0.60 | 0.037 | 0.53-0.68 | <0.001 |
| Without social support | 0.38 | 0.053 | 0.28-0.49 |  |

^a^ Adjusted Wald test to detect differences in predicted probabilities within each BMI group.
